# Culture-free genotyping of *Neisseria gonorrhoeae* revealed distinct strains at different anatomical sites in a quarter of patients, the Netherlands, 2012 to 2016

**DOI:** 10.2807/1560-7917.ES.2018.23.50.1800253

**Published:** 2018-12-13

**Authors:** Brian MJW van der Veer, Petra FG Wolffs, Christian JPA Hoebe, Nicole HTM Dukers-Muijrers, Lieke B van Alphen

**Affiliations:** 1 Department of Medical Microbiology, Care and Public Health Research Institute (CAPHRI), Maastricht University Medical Centre (MUMC+), Maastricht, the Netherlands; 2 Department of Sexual Health, Infectious Diseases and Environmental Health, South Limburg Public Health Service, Heerlen, the Netherlands

**Keywords:** Neisseria, gonorrhoeae, genotyping, surveillance, concurrent infections

## Abstract

**Background:**

Genotyping of *Neisseria gonorrhoeae* (NG) is essential for surveillance to monitor NG transmission and dissemination of resistant strains. Current genotyping methods rely on bacterial culture which frequently fails.

**Aim:**

Our aim was to develop a culture-free genotyping method that is compatible with the widely used *N. gonorrhoeae* multi-antigen sequence typing (NG-MAST) database, which facilitates genotyping of NG detected at separate anatomical sites in individual patients.

**Methods:**

Specific primers for both PCR targets *porB* and *tbpB* were designed and technically validated by assessing the analytical sensitivity, cross-reactivity with 32 non-gonoccocal *Neisseria* species, and concordance with NG-MAST. Clinical application was assessed on 205 paired samples from concurrent NG infections at different anatomical sites of 98 patients (81 men who have sex with men and 17 women) visiting our sexually transmitted infections clinic.

**Results:**

Typing could be consistently performed on samples with a PCR quantification cycle (Cq) value <35. Furthermore, the method showed no cross-reactivity and was concordant with NG-MAST. Culture-free NG-MAST improved the typing rate from 62% (59/95) for cultured samples to 94% (89/95) compared with culture-dependent NG-MAST. Paired samples of 80 of 98 patients were genotyped, revealing distinct NG strains in separate anatomical sites in 25% (20/80) of the patients.

**Conclusions:**

This NG-specific genotyping method can improve NG surveillance as it facilitates genotyping of non-culturable and extra-genital samples. Furthermore, 25% of patients were infected with multiple NG strains, which is missed in current culture-dependent surveillance. Including non-culturable and concurrent NG infections in surveillance informs actions on dissemination of multidrug-resistant NG strains.

## Introduction

*Neisseria gonorrhoeae* (NG) is one of the most common bacterial sexually transmitted infections [1]. The World Health Organization (WHO) estimates that more than 100 million new cases of NG occur each year, even though testing for NG and diagnostics have improved [1,2]. Detection of NG allows empirical treatment that results in cure in at least 95% of cases, and rapid cure subsequently limits transmission [1]. However, increasingly resistant strains of NG have been reported in the last decades, which could complicate empirical treatment [3]. Therefore, gaining insight in transmission and antimicrobial resistance (AMR) of NG is important. NG can be detected by culture or nucleic acid amplification test (NAAT) but both methods have limitations [2]. Culture is known to be less sensitive because NG requires demanding nutritional and environmental conditions, leading to a low percentage of culture-confirmed diagnoses [4]. In contrast, NAAT are more sensitive but cannot determine the AMR profile [2,5].

Surveillance of NG is essential to monitor transmission and dissemination of resistant strains. NG multi-antigen sequence typing (NG-MAST) is a widely used genotyping method to monitor transmission and outbreaks [6,7]. This method has a higher discriminatory power than multilocus sequence typing (MLST) and is more cost-effective than highly discriminatory whole genome sequencing [8,9]. In addition, some of the NG-MAST sequence types (ST) are associated with AMR [7,10]. The currently used NG-MAST protocol requires culture because the primers cross-react with other *Neisseria* species [11]. To date, only two studies have genotyped non-cultured clinical samples with NG-MAST [6,11]. Whiley et al. demonstrated that NG-MAST can be applied to non-cultured urogenital samples but not to samples from extra-genital sites (oropharynx and rectum) because of the presence of commensal *Neisseria* species [11]. They showed that mainly *N. lactamica*, *N. meningitidis* and *N. polysaccharea* strains lead to the cross-reactivity. Furthermore, it appeared that successful application of NG-MAST to non-cultured samples was linked to the quantification cycle (Cq) of the PCR-positive sample because four of the five failed samples had a high Cq value (>35).

Previous studies have shown that patients can be NG-positive at extra-genital sites and have concurrent NG infections at different anatomical sites [12,13]. Most of the extra-genital and concurrent infections are observed in risk groups, for example in men who have sex with men (MSM). Extra-genital sites may act as a reservoir for AMR genes as the present commensal *Neisseria* species, potentially harbouring AMR genes, readily exchange DNA with NG [14]. Typing the oropharyngeal site using culture-dependent methods is especially difficult because the bacterial load is lower than at other anatomical sites and this appears to be linked to culture success [15]. In previous studies, concurrent NG infections were studied with various genotyping methods [6,11,16-18]. Distinct NG strains per anatomical site have been observed and some strains demonstrated discordant antibiotic susceptibility profiles [6,16-18]. The observed distinct NG strains could be explained by high-risk sexual behaviour and patients being part of different transmission chains [16,18]. However, these studies were small (fewer than 10 patients), focussed on cultured isolates, used a single-position single nucleotide polymorphisms (SNP) and/or used non NG-specific primers [6,16-18]. Therefore, we aimed to develop a culture-free NG-MAST genotyping method that does not cross-react with other *Neisseria* species and is compatible with the NG-MAST database. Furthermore, we aimed to gain more insight in the frequency of distinct NG strains at separate anatomical sites in individual patients.

## Methods

This study was designed to test the clinical application of the culture-free NG-MAST method to non-culturable clinical samples and use these data to compare ST of separate anatomical sites within a patient. The method was technically validated by assessing analytical sensitivity, specificity and concordance with NG-MAST.

### Clinical samples

All NG-positive clinical samples (n = 1,110) from different anatomical sites were retrieved from 814 consultations (further referred to as number of patients) from 642 individual patients. NG positivity was based on NG detection by the Cobas 4800 CT/NG NAAT assay (Roche Diagnostics, Basel, Switzerland), between January 2012 and May 2016 from our sexually transmitted infections (STI) clinic (South Limburg Public Health Service). These samples were from MSM (n = 769 samples), women (n = 254 samples) and heterosexual men (n = 87 samples). Samples with a Cq value of 35 or higher did not consistently yield PCR products in dilution series (see technical validation). Therefore, clinical samples with a Cq value of ≥35 were excluded (n = 418), leaving 692 samples for analysis. Of the remaining 692 NG-positive samples, we included only paired samples from separate anatomical sites belonging to a single STI clinic visit of a patient (n = 228). Different pairs of any combination of genital, anorectal or oropharyngeal NG positivity were observed. A total of 108 patients were NG-positive at two or three anatomical sites (90 MSM and 18 women who reported anal sex or symptoms and who were systematically tested on all three anatomical sites). The remaining amount of sample material was not sufficient for typing for 10 patients (nine MSM and one woman) and therefore these patients were excluded, leaving 98 patients with paired samples for analysis. In total, 205 Cobas NAAT clinical samples were included: 57 urine, 17 vaginal, 92 anorectal and 39 oropharyngeal samples. With these samples, we assessed the clinical application of the culture-free NG-MAST method and the presence of distinct STs within a patient. Data on culture success were retrieved by routine diagnostics because NG culture is mostly performed as part of the national NG resistance surveillance since NAAT diagnosis of NG is the primary diagnostic procedure. All patients were treated with a single dose of ceftriaxone, the primary choice of treatment because no resistance exists in the Netherlands [4]. An additional swab or urine sample for routine NG culture is taken at the treatment visit at the STI clinic only when treatment has not already been provided at the diagnostic visit based on symptoms. For this study, data of this routine culture was available for all patients if culture was performed.

### DNA isolation clinical samples

Total DNA was isolated from 400 µL Cobas 4800 clinical samples using the QIAamp DNA Mini Kit (Qiagen, Hilden, Germany) and eluted in 50 µL Milli-Q water (MQ). To increase elution yield, we extended the incubation time to 10 min. The eluate was stored at −20 °C.

### DNA isolation cultured gonoccocal and non-gonoccocal *Neisseria* strains

Gonoccocal and non-gonoccocal clinical and reference *Neisseria* strains were inoculated on chocolate agar with IsoVitaleX or blood agar (BectonDickinson, Sparks, United States (US)) and incubated over night at 37 °C in 5% CO_2_. Morphology of the colonies was checked and a single colony was subcultured before DNA isolation. Bacterial suspensions were prepared in sterile saline solution from two or three colonies (depending on the size of the colonies) picked with a pre-wetted sterile swab. The bacteria were pelleted by centrifugation at 2,000 g for 5 min and washed once. The pellet was resuspended in 500 µL MQ and boiled for 10 min. Cell debris was pelleted by centrifugation at 8,000 g for 2 min and the supernatant was stored at −20 °C.

### NG-MAST genotyping

PCR for both targets was performed in 50 µL reaction volumes using the Biometra T3000 Thermal Cycler (Labrepco Inc., US). Each reaction per target (*porB* and *tbpB*) contained 50 pmol of the NG-MAST forward and reverse primer for the respective target (Table 1), 2.5 U HotStar polymerase (Qiagen), 1× Qiagen PCR buffer, 0.2 mmol/L dNTP, 5 µL DNA lysate and MQ to a volume of 50 µL. The PCR protocol of Martin et al. was used to amplify the targets but cycles were increased to 30 [7].

**Table 1:** Overview of primers used in PCR and sequencing reactions for NG-MAST and culture-free NG-MAST

	NG-MAST [7]	Culture-free NG-MAST
PCR primers *porB* Forward Reverse	5’-CAA GAA GAC CTC GGC AA-3’ 5’-CCG ACA ACC ACT TGG T-3’	5’-GTT AAT CCG CTA TAA CCC CC-3’ 5’-CCG ACA ACC ACT TGG T-3’
PCR primers *tbpB* Forward Reverse	5’-CGT TGT CGG CAG CGC GAA AAC-3’ 5’-TTC ATC GGT GCG CTC GCC TTG-3’	5’-TTC CTT CCA AAA AAC CGG AAG CCC G-3’ 5’-CAT TGC CCG GAT AGG CAA ACC A-3’
Sequence primers *porB* Forward Reverse	5’-CAA GAA GAC CTC GGC AA-3’ 5’-CCG ACA ACC ACT TGG T-3’	5’-CAA GAA GAC CTC GGC AA-3’ 5’-CCG ACA ACC ACT TGG T-3’
Sequence primers *tbpB* Forward Reverse	5’-CGT TGT CGG CAG CGC GAA AAC-3’ 5’-TTC ATC GGT GCG CTC GCC TTG-3’	5’-CGT TGT CGG CAG CGC GAA AAC-3’ 5’-TTC ATC GGT GCG CTC GCC TTG-3’

The amplicons were precipitated with 50 µL 20% polyethylene glycol 8000 and 2.5 mol/L sodium chloride at 37 °C for 15 min. Precipitated amplicons were centrifuged at 15,000 × g for 15 min and washed twice with ice-cold 80% ethanol. The pellet was allowed to dry and resuspended in 25 µL MQ.

The *porB* and *tbpB* fragments were sequenced with their respective forward and reverse primer using the BigDye Terminator v1.1 Cycle Sequencing kit (Thermo Fisher Scientific, Waltham, Massachusetts, US). The sequence protocol has an initial denaturation step of 1 min at 96 °C, followed by 25 cycles of 10 s at 96 °C, 10 s at 55 °C (*porB*) or 65 °C (*tbpB*), and 3 min at 60 °C.

### Primer design culture-free NG-MAST

The genome sequences of all NG reference strains published by the WHO (n = 14) were downloaded from GenBank and used for multiple alignments with Clustal Omega [19]. A 2 kb flanking region of the aligned NG-MAST *porB* and *tbpB* primers were selected to identify conserved regions. Each flanking region was aligned and conserved regions were tested for in silico specificity using basic-local alignment search tool (BLAST). A specific sequence was identified that could be used as the forward primer for *porB* but no specific sequence was identified for the reverse primer, therefore the NG-MAST reverse primer was used which resulted in a fragment of ca 1.2 kb (Table 1). Two specific sequences were identified for *tbpB* which could be used as a forward and reverse primer, resulting in a fragment of ca 1.8 kb (Table 1).

### Culture-free NG-MAST genotyping

This method was similar to the NG-MAST method apart from the initial PCR. Each reaction per target (*porB* and *tbpB*) contained 50 pmol of the culture-free NG-MAST forward and reverse primer for the respective target (Table 1), 0.2 µL AccuPrime Taq DNA Polymerase High Fidelity (Thermo Fisher), 1× AccuPrime PCR buffer II, 15 µL DNA isolated from a clinical sample and MQ to a volume of 50 µL. The PCR protocol had an initial denaturation step of 5 min at 95 °C, followed by 40 cycles of 30 s at 95 °C, 60 s at 58 °C (*porB)* or 69 °C (*tbpB*), 2.5 min at 68 °C, and a final extension of 10 min at 68 °C. The *porB* and *tbpB* amplicons were sequenced with NG-MAST primers (Table 1). The culture-free method was therefore compatible with the NG-MAST online database because we characterised the same fragments of *porB* and *tbpB* genes.

### Technical validation of culture-free NG-MAST method

Analytical sensitivity was determined using dilution series ranging from 1.3 × 10^6^ to 1.3 × 10^2^ colony-forming units (CFU)/mL. Concordance of culture-free NG-MAST method with NG-MAST was tested with seven randomly selected isolates cultured from four urine samples, two anorectal swabs and one oropharyngeal swab, and their respective unculturable Cobas 4800 screening samples between January and March 2017. The isolates were subjected to NG-MAST genotyping whereas the clinical samples were subjected to the culture-free NG-MAST method. The analytical specificity was tested with a panel of 32 non-gonococcal *Neisseria* species strains, including *N. cinerea* (n = 1), *N. denitrificans* (n = 1), *N. elongata* (n = 1), *N. flavescens* (n = 1), *N. lactamica* (n = 2), *N. meningitidis* (n = 3), *N. mucosa* (n = 7), *N. perflava* (n = 1), *N. polysaccharea* (n = 1) and *N. subflava* (n = 14).

### Data analysis

The trace files were assembled, trimmed and edited using Bionumerics (version 7.6, Applied Maths, Sint-Martens-Latem, Belgium). The starting trimming patterns for *porB* and *tbpB* and lengths were used as described in Martin et al. [7]. Alleles and ST were called according to the NG-MAST online curated database. Phylogenetic trees of *porB*, *tbpB* and concatenated sequences were constructed using multiple alignment and unweighted pair group method with arithmic mean (UPGMA) clustering using default settings with gap penalty at 100%.

### Ethical statement

The study protocol was approved as a scientific study not done in humans by the Medical Ethical Committee of Maastricht University Medical Centre (MUMC+; number METC 2017–2-0251) as it concerned a laboratory and observational study using anonymous data and leftover diagnostic samples only. This was part of an STI clinic procedure where patients did not object to the use of their data and samples anonymously for research purposes.

## Results

### Analytical sensitivity, specificity and concordance of culture-free NG-MAST

Dilution series in triplicate showed that culture-free NG-MAST consistently yielded PCR products for both *porB* and *tbpB* in samples with a Cq value <35. None of the tested 32 non-gonococcal *Neisseria* strains were PCR-positive for either *porB* or *tbpB* in the culture-free NG-MAST PCR reactions. The seven randomly selected cultured isolates had identical ST as their respective unculturable Cobas 4800 screening sample but distinct ST were observed between the selected isolates.

### Paired clinical samples

In total, 90.2% (185/205) of the selected paired clinical samples were successfully genotyped with the culture-free NG-MAST method. The *porB* fragment was successfully sequenced in 95.6% (196/205) of samples and *tbpB* in 93.7% (192/205). Failure of both targets in a sample does not appear to be related to the Cq value because both low (<30) and higher (30–35) Cq values show comparable failure rates (data not shown). We observed 36 different *porB* and 22 *tbpB* alleles, resulting in 45 ST. Among the samples, *porB*-1808 and *tbpB*-29 were the most common alleles, present in 51 and 49 samples, respectively. Furthermore, we found five previously unidentified *porB* and two *tbpB* alleles which all had the highest identity with NG using a BLAST search. The most prevalent ST were ST2992 (n = 36), ST11461 (n = 30), and ST5441 (n = 26), and 15 new STs were found.

Routine culture was performed for 95 of the 205 paired clinical samples and 59 (62.1%) were culture-positive. Typically, only one anatomical site was sampled for culture, and the majority of the culture-positive samples were collected from the genital site (44/59). Culture-free NG-MAST applied to the non-culturable clinical material (Cobas 4800 sample material) of samples send in for culture (including culture-negative samples) showed that 93.7% (89/95) were genotyped successfully. However, four samples negative in culture-free NG-MAST were culture-positive. Of the remaining 110 uncultured clinical samples, 98 (89.1%) could be genotyped.

### Sequence diversity within *porB* and *tbpB* alleles

High sequence diversity was observed for both *porB* and *tbpB* in this study population (Supplement Figures S1 and S2). Two *porB* alleles (90 and 2723) were divergent, with more than 50% dissimilarity, from all other observed alleles. The newly identified *tbpB* allele with 91% similarity with *tbpB*-1251 was divergent from all other observed alleles with more than 60% dissimilarity. In addition, the average dissimilarity between *tbpB* alleles appeared to be greater than between *porB* alleles.

### Sequence types of samples from separate anatomical sites in a patient

In this dataset of clinical samples, we genotyped 169 paired samples (taken from a single patient at separate anatomical sites) from 80 patients (66 MSM and 14 women) (Supplement Table). We observed distinct concurrent ST in a quarter (20/80) of the patients. They had the following combinations of sample material: urine-anorectal (n = 6), urine-oropharyngeal (n = 1), anorectal-oropharyngeal (n = 8), urine-anorectal-oropharyngeal (n = 1), vaginal-anorectal (n = 3), and vaginal-anorectal-oropharyngeal (n = 1) (Table 2). Similar proportions of distinct concurrent ST were observed in MSM (16/66) and women (4/14). Interestingly, a single patient (patient 32) was NG-positive with a distinct NG strain at all three tested anatomical sites (Table 2).

**Table 2:** Characteristics of patients with concurrent *Neisseria gonorrhoeae* infection with distinct sequence types, including age, risk group, multiple sequence types and NG-MAST results per sample site, the Netherlands, January 2012–May 2016 (n = 42)

Patient	Age (years)	Risk group	Urine			Vaginal			Anorectal			Oropharyngeal		
			*porB*	*tbpB*	ST	*porB*	*tbpB*	ST	*porB*	*tbpB*	ST	*porB*	*tbpB*	ST
9	26	Women^a^	-	-	-	1808	91%-1251	New ST1	1808	29	2992	-	-	-
10	22	MSM	-	-	-	-	-	-	3031	33	4995	1489	33	10257
24	27	MSM	-	-	-	-	-	-	7272	33	New ST4	99%-7988	110	New ST3
27	24	Women^a^	-	-	-	301	29	359	2723	110	4431	301	29	359
28	20	MSM	-	-	-	-	-	-	908	27	3588	2723	27	15046
29	41	MSM	6720	188	11461	-	-	-	1808	137	11084	-	-	-
31	44	MSM	7988	110	13902	-	-	-	908	110	1407	-	-	-
32	42	MSM	182	74	1247	-	-	-	1808	29	2992	1808	836	New ST6
33	21	Women^a^	-	-	-	99%-6405	74	New ST7	6720	188	11461	-	-	-
37	25	MSM	1808	188	New ST8	-	-	-	182	74	1247	-	-	-
39	20	MSM	-	-	-	-	-	-	3031	33	4995	4288	4	9382
48	21	MSM	-	-	-	-	-	-	6720	188	11461	4288	188	New ST9
53	41	MSM	30	18	5441	-	-	-	3059	29	5049	-	-	-
54	25	MSM	-	-	-	-	-	-	1808	29	2992	30	18	5441
55	36	Women^a^	-	-	-	1808	29	2992	6720	188	11461	-	-	-
58	49	MSM	30	18	5441	-	-	-	-	-	-	1808	29	2992
59	26	MSM	-	-	-	-	-	-	1808	29	2992	30	18	5441
60	42	MSM	30	18	5441	-	-	-	1808	29	2992	-	-	-
79	20	MSM	-	-	-	-	-	-	4199	29	New ST14	4199	4	New ST12
80	19	MSM	6720	188	11461	-	-	-	30	29	298	-	-	-

NG-MAST: *Neisseria gonorrhoeae* multi-antigen sequence typing; ST: sequence type.

^a ^Women reporting anal sex or symptoms.

Sample material that was NG-negative or not sampled as part of routine diagnostics is indicated with a hyphen.

The Figure presents the dissimilarity of concatenated sequences of *porB* and *tbpB* between STs. For the majority of the patients with distinct concurrent STs, a large (>15%) dissimilarity was observed between the concatenated sequences. Patients 31 and 48 had only 1% dissimilarity between the concatenated sequences. In both patients, the *tbpB* allele was identical between the distinct ST but the *porB* allele showed >1% dissimilarity, meaning the two ST did not belong to the same genogroup. When assigning ST to genogroups, we identified two genogroups that consisted of more than five samples: G2992 (n = 32) and G11084 (n = 6). Furthermore, we identified three samples belonging to G1407 (one ST1407 and two ST2212) of which only one (ST2212) was culture-positive and susceptible for ceftriaxone.

**Figure:**
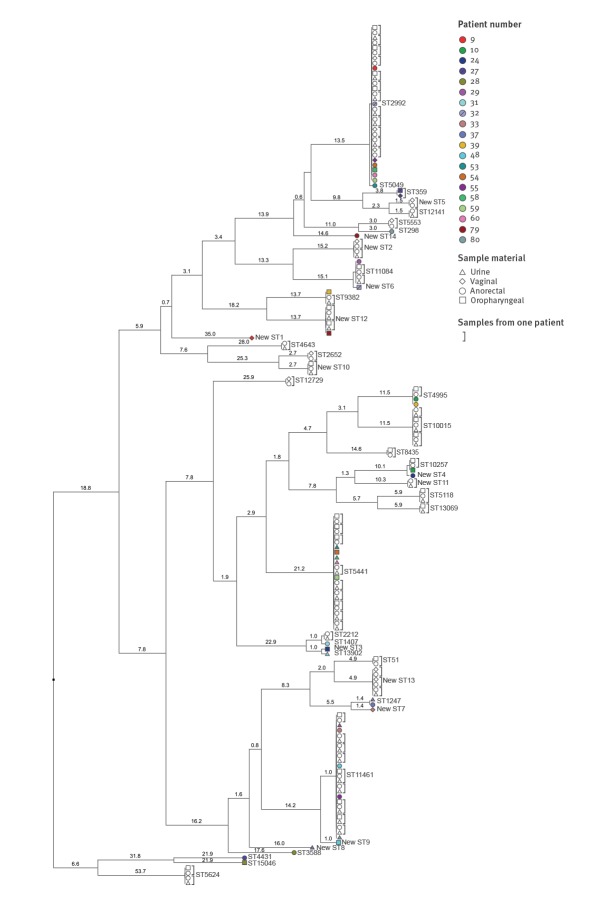
Dendogram constructed by multiple alignment of concatenated *porB* and *tbpB* sequences clustered with unweighted pair group method with arithmic mean (UPGMA) algorithm, the Netherlands, January 2012–May 2016 (n = 169)

Sequence type (ST) of each sample is shown at the tips of the dendogram and samples of patients with distinct ST are coloured. The branch length depicts the percentage of dissimilarity.

## Discussion

In this study we show that the culture-free NG-MAST method can readily be used to genotype NG in clinical samples including extra-genital samples. In addition, the method is compatible with the online NG-MAST database. The culture-free NG-MAST method was technically validated by assessing the NG analytical specificity using non-gonoccocal *Neisseria* species; it demonstrated good specificity for NG as no cross-reactivity was observed. Furthermore, concordance with NG-MAST was demonstrated by comparing typing results of non-culturable clinical samples (Cobas 4800 sample material) with cultured isolates, which were taken less than 2 weeks apart. In this time frame, identical genotypes were expected based on the study by Martin et al. [7]. With culture-free NG-MAST, we genotyped 90% of the selected paired clinical samples with sufficient bacterial load (Cq value <35). Extrapolating this genotyping rate to all NG positive samples (n = 1,110) would result in successful typing of 56% (624/1,110) of all NG-positive samples. Among all samples sent in for culture, culture-free NG-MAST showed a higher typing rate of 94% (89/95) compared with the culture-dependent method with 62% (59/95). However, four of the 59 culture-positive samples were negative in culture-free NG-MAST. The clinical samples testing negative in culture-free NG-MAST could be caused by PCR-inhibitory substances in the clinical material. The majority of the culture-positive samples were collected from the genital site (44/59); that could be explained by the sampling strategy, but the low sensitivity of extra-genital NG culture could also have contributed [2]. This highlights the importance of culture-free genotyping as the current surveillance data would be biased towards genital samples. With our method, we were able to genotype 33 of the 36 culture-negative samples, which were mainly extra-genital samples (25/33).

Our results show that both sexes were frequently infected with distinct NG strains in a quarter of patients (20/80) which is higher than most previous studies [6,11,17,18]. The studies of Whiley et al. (0/4) and Carannante et al. (1/8) assessed, respectively, only four and eight patients with paired samples, which could explain the lower proportion [6,11]. Pond et al. (3/71) developed a real-time PCR assay to predict ciprofloxacin resistance with the detection of a resistance-associated SNP [17]. This method uses a single position to identify distinct strains of NG, leading to a lower resolution than NG-MAST where two internal fragments (490 bp and 390 bp) of highly polymorphic genes are analysed. De Silva et al. (26/206) performed whole genome sequencing only on cultured strains and therefore may have missed distinct strains from samples that were NAAT-positive but culture-negative [18]. A higher percentage of distinct strains in paired clinical samples was reported in a study by Kolader et al. (52/130) which applied *por*-*opa* restriction fragment length polymorphism typing [16]. The authors hypothesised that the observed high frequency could be the result of high-risk sexual behaviour or also of recombination in the *opa* genes.

High-risk behaviour and sex with multiple sex partners on the same occasion may explain the frequently observed distinct ST in our study as we included MSM and women reporting anal sex or symptoms attending our STI clinic, who are considered as risk groups [1,16]. Another possible reason could be DNA exchange with commensal *Neisseria* species or other NG strains [11,16]. Patients colonised with multiple NG strains could have different AMR profiles, potentially resulting in under-treatment which could allow dissemination of resistant strains [6,17]. However, the impact of multiple strain infections on treatment needs to be addressed in future research to answer questions of the effect on AMR development and dissemination of resistant strains.

The observed concurrent infections with distinct strains in our study would be overlooked in routine diagnostics as Dutch and European (European Centre for Disease Prevention and Control) NG resistance surveillance guidelines recommend culture of only one anatomical site [2,20,21]. Without typing data for concurrent NG infections, surveillance data are incomplete and potential transmission links or associations between ST and AMR can be missed. This potentially results in dissemination of unrecognised resistant types. Therefore, early detection and improved surveillance of ST that are linked to AMR could minimise sequelae and prevent dissemination of multidrug-resistant strains.

We observed high variability in both alleles and ST in our study population, which could be due to sampling over a prolonged time period and from different risk groups (MSM and women reporting anal sex or symptoms). For example, ST2212, ST2992, ST5441 and ST5793 are more prevalent in MSM than heterosexual men or women [10,22,23]. In our study, these STs were mainly found in MSM, but eight samples with ST2992 and one with ST5793 were from women. Interestingly, we found three samples which belong to the genogroup G1407 (ST1407 (n = 1) and ST2212 (n = 2)) linked to decreased susceptibility to the last first-line treatment with ceftriaxone [10,23]. Only one of the three could be cultured (ST2212) and was susceptible to ceftriaxone. Furthermore, ST359, ST2992, ST3588 and ST4995 are linked to azithromycin resistance which is the recommended dual-therapy treatment with ceftriaxone in case of a *Chlamydia trachomatis* co-infection [1,23-25]. In addition, this dual therapy is applied to slow down emerging resistance or where local resistance data are not available [1,5,20]. In the Netherlands, a single treatment with ceftriaxone is applied because no resistance has yet been found in the Netherlands [4]. However, a multidrug-resistant isolate was recently found in the United Kingdom that showed high-level resistance to both ceftriaxone and azithromycin, thereby highlighting the need for improved surveillance [26]. In our study population, we found a high prevalence of ST belonging to genogroup G2992 (19.5%), which is in line with earlier data from the Netherlands (16.1%), while genogroups G1407 (1.7%) and G359 (1.1%) were less frequent (respectively 7.7% and 6.3% in the study of Wind et al.) [23]. The genogroup G2992 is also frequently observed in most other countries in Europe [27] and G1407 prevalence is higher in most European countries than in the Netherlands.

Even though many NG-MAST ST are linked to resistance profiles in NG, this does not necessarily imply that the strain is phenotypically resistant [7,10]. Additional tests that can identify mutations leading to resistance, for example azithromycin resistance, could give more insight into those strains that cannot be cultured [28]. A limitation of this study is that we only included samples with a higher NG load (Cq value <35); therefore the typing rate of samples with a lower bacterial load is unknown. However, as culture success is also associated with bacterial load, culture-dependent methods are expected to perform worse than our culture-free method in samples with a low NG load (Cq value ≥35). This hypothesis is strengthened because only 10% (18/188) of the samples with a Cq value ≥35 were culture-positive in routine diagnostics. A nested PCR approach might improve genotyping of samples with a low bacterial load as has been applied for medico legal purposes to allow typing from a piece of clothing [29].

### Conclusion

The culture-free NG-MAST method can genotype NG from most non-culturable clinical samples, including extra-genital samples, as cross-reactivity with commensal *Neisseria* species was not observed. Compared with culture-dependent NG-MAST, culture-free NG-MAST has a higher typing rate and does not have high demands on sample conditions. Applying culture-free NG-MAST to clinical samples revealed frequent concurrent infections with distinct ST at separate anatomical sites in MSM and women reporting anal sex or symptoms. These distinct concurrent ST and extra-genital NG infections would be missed in the current European surveillance strategy possibly allowing dissemination of resistant NG strains. Including non-culturable and concurrent NG infections in surveillance informs actions to contain the dissemination of multidrug-resistant NG strains.
